# The L.C.C.'s Employment of Uncertified Nurses

**Published:** 1919-10-11

**Authors:** Gladys M. E. Leigh


### The L.C.C.'s Employment of Uncertified Nurses.[1]

*To the Editor of* The Hospital.

Sir,—In a current advertisement, the Clerk of the London County Council invites applications for the position of nurse at the Upton House Industrial School, Homerton, N.E. The salary, including a war bonus, plus temporary additional remuneration due to economic conditions, commences at £51 15s. per annum, together with the usual allowances. The advertisement states applicants must have received hospital training, but need *not* necessarily hold the certificate granted after a course of three years' training. Applications for forms are to be made to the Educational Officer, Education Offices, W.C. 2. Briefly, this advertisement, encouraging the employment of partially trained nurses, bears the official stamp. Moreover, if it has been inserted with the cognisance and authority of the Ministry of Health, it is a matter of vital importance to members of the nursing services. It is an acknowledged fact that during the years of war health visitors were appointed with no higher qualifications than the C.M.B. certificate, but the emergency which rendered advisable these appointments happily no longer exists. Only a month since we were told that there were still 1,500 certificated women who had served in the Q.A.I.M.N.S.R. and T.F.N.S. without civilian posts, and yet the L.C.C. apparently is willing and able to ignore their need and to further encourage the dilution of the nursing services with women who have failed to obtain the certificate granted by a recognised training-school. Surely the hour has struck for the Ministry of Health to state, and to state clearly, the value at which it appraises the certificates granted by the training-schools of Great Britain, and to define without loss of time the future official status it proposes to accord to the trained certificated nurses in the life of the community.—Yours faithfully,

Gladys M. E. Leigh.

